# Animal Lives Affected by Meat Consumption Trends in the G20 Countries

**DOI:** 10.3390/ani14111662

**Published:** 2024-06-01

**Authors:** Sytske van der Laan, Gerard Breeman, Laura Scherer

**Affiliations:** 1 Institute of Environmental Sciences (CML), Leiden University, 2333 CC Leiden, The Netherlands; 2 Institute of Public Administration, Leiden University, P.O. Box 13228, 2501 EE Den Haag, The Netherlands

**Keywords:** animal welfare, animal-source foods, livestock, temporal development, forecast, animal farming governance

## Abstract

**Simple Summary:**

Animal-source foods, such as meat, inevitably affect the animals used for their production. The amount of animal-source foods consumed also influences how many individual animals are affected. Globally increasing consumption can have serious consequences for animal welfare. This study aims to quantify how the number of animals affected by meat consumption has developed over time in the past decades within the G20 countries and how it is likely to develop further by 2030. The number of animal lives affected is increasing due to increasing meat consumption, but the size of the changes differs among countries and animal categories. The number of animals affected increases faster in emerging countries, such as China, than in more industrialized countries, such as Germany. Poultry birds, which yield less meat per animal, are affected by far the most. A shift towards even more poultry implies that the number of animals affected grows substantially faster than meat consumption. Until 2030, we can expect further increases in the number of animals affected due to the increasing meat consumption and share of poultry meat. The findings highlight the need for stronger regulations and a more informed and conscientious global dialogue to steer food systems toward a more sustainable and animal-friendly future.

**Abstract:**

Trends in dietary habits have far-reaching implications, but their impact on animals remains insufficiently explored, as many people continue to dissociate meat from individual animal lives. This research study quantifies the temporal development of the number of animal lives affected by meat consumption within the G20 countries between 1961 and 2020 and forecasts for 2030. Production (including slaughter) and historical and projected food balance data were analyzed to explore these trends. The results indicate an increase in the number of animal lives affected due to increasing consumption, but discrepancies exist between different countries and animal categories. Increases are stronger in emerging countries, such as China, than in more industrialized countries, such as Germany. Overall, the number of animals affected grows 1.7 times as fast as meat consumption due to a shift towards poultry. Poultry birds are affected by far the most, and their dominance in number only slightly reduces when considering the differentiated moral values of the animals, reflecting their sentience. Until 2030, we can expect further increases in the number of animal lives affected. The findings highlight the need for progressive legislation to address the complex trade-offs and challenges in reversing the increasing trends in the number of animals affected.

## 1. Introduction

“We are not ostriches, and cannot believe that if we refuse to look at what we do not wish to see, it will not exist. This is especially the case when what we do not wish to see is what we wish to eat” [1]. This discerning perspective summarizes the fundamental challenge underlying the global transition toward a more animal-friendly diet. It highlights the dissociation between meat consumption and the individual animals slaughtered for it [2].

The relationship between humans and animals has evolved noticeably over time, from early domestication for purposes of sustenance and labor to a complex interplay of cultural, economic, and ethical dimensions in modern society [3]. As meat consumption continues to surge worldwide, farmers try to increase productivity and maximize profits by switching from small-scale to intensive animal farming [4]. Consequently, questions regarding the ethical treatment and well-being of the animals involved have become increasingly pressing. Animal welfare concerns basic health and functioning, affective states (emotional experiences), and natural behavior [5]. Most livestock are slaughtered after a few weeks or months, only 2 to 20% of their normal life span [6]. Livestock is defined as domesticated animals raised in an agricultural setting to provide labor and produce diversified products for consumption, such as meat, eggs, milk, fur, leather, and wool [7]. The livestock industry often involves various harmful (and partly even legalized) practices. Examples include challenges faced by animals bred in overcrowded and confined systems; mutilations without anesthesia; the stressful conditions of transportation potentially resulting in injuries or fatalities among animals; the prolonged fear, pain, and respiratory distress animals experience before slaughter; and slaughter without stunning [6,8]. These conditions have been criticized by public health professionals and animal welfare advocates [9].

Despite increasing ethical concerns for livestock animals, global meat consumption trends do not portray a brighter future for animals destined to end up on our plates. The global demand for meat consumption has reached unprecedented levels in recent decades, driven by factors such as population growth, rising affluence, and changing dietary preferences [10]. Studies have shown that these numbers will most likely continue to increase as a consequence of the growing demand for meat [11]. Trends in dietary habits have far-reaching implications for various aspects of human society, including public health, environmental sustainability, and economic systems [12]. However, the aspect of this phenomenon that remains insufficiently explored, despite increasing attention, is its profound impact on animals and animal welfare. Extensive research exists on trends in meat consumption, along with a robust scholarly discussion surrounding the growing ethical concerns for animal welfare. However, because of the above-mentioned dissociation between meat and animals, there is little awareness in both academia and society of what consumption trends mean for the number of individual animal lives affected.

To address this gap observed within the scientific discourse, this study couples meat consumption trends with a comprehensive examination of affected animals. The aim of the research is to make the animal lives affected more explicit by quantifying the individual lives of animals embodied in meat consumption. Therefore, the central question guiding this study is as follows: How many individual animal lives are affected by the trends in meat consumption within the G20 countries in the past and the future? A follow-up question is as follows: How does a moral value, reflecting the animals’ sentience, change the contributions from different animal categories affected by the trends in meat consumption? Finding the answer to these questions aims to provide a foundation for future discussions and actions that prioritize both humans and animals within a sustainability framework.

Closing this knowledge gap is of societal relevance, as we often try to dissociate meat from animals, even if we feel compassion for them [2]. The hope is that this study prompts readers to consider not just the human aspects of the meat industry but also the individual animals slaughtered for it. Creating more awareness amongst consumers, animal welfare advocates, and policymakers about the number of animals affected can help motivate them to make and promote (e.g., through legislation) more animal-friendly choices. We will also discuss what animal welfare legislation could reverse trends in the number of animals affected and mitigate the impact on individual animals. Additionally, closing the knowledge gap is of scientific relevance, as academia currently lacks a deeper understanding of animals’ lives affected by human consumption trends. By providing a tangible overview of the consequences of global meat consumption trends on the number of individual animal lives, this study aims to contribute to a more informed and conscientious dialogue on the ethics and sustainability of dietary choices, urging the consideration of animal welfare within a sustainability framework.

## 2. Materials and Methods

### 2.1. Data Collection

This study focused on the nineteen countries comprising the G20 to ensure that a collection of both developed and emerging economies is represented in the analysis (Table A1). The G20 countries account for 79% of the global gross domestic product, 59% of the world’s population, and 73% of the global caloric meat supply in 2021 [13]. They thus provide a broad representation of the world in relation to meat supply. The European Union and African Union were excluded from the analysis, as they are supranational organizations representing the aggregated demographic, political, and economic profiles of multiple member states. Analyzing the G20 countries individually allows for more detailed cross-country comparisons. The categorization of countries used in the analysis adhered to the framework outlined by the G20. This framework organizes countries into five groups [14]. The allocation within these groups predominantly followed regional affiliations [14]. 

The Food and Agriculture Organization (FAO) was the main source of data required to conduct the quantitative analysis. The FAO collects data on food and agriculture for over 245 countries, including the G20 countries, from 1961 to the most recent year available (at the time of this study and the factors analyzed, that was the year 2020) and shares this through its statistical database FAOSTAT [13]. These datasets are indispensable to the analysis of animal lives affected and meat consumption research. The four largest meat categories that FAOSTAT provides consumption data for (Table A2) were also used for further purposes of this study: (1) bovine meat, (2) pigmeat, (3) poultry meat, and (4) mutton and goat meat [13]. Different elements sourced from FAOSTAT were used for the analysis (Table A5). Before analyzing the data, certain adjustments were necessary to refine the different datasets. Integration of two datasets was necessary due to FAOSTAT’s segregation of information based on distinct methodologies employed before and after 2010. Also, most data from FAOSTAT were expressed in carcass weight equivalent (CWE) but were converted to meat in boneless retail weight equivalent (RWE) for this study. Retail weight reflects the weight as it is sold to consumers, excluding bones and organs [13]. Data were converted because retail weight more closely represents the actual meat consumed at the national level. Additionally, since poultry meat values were already provided in ready-to-cook weight, which closely approximates retail weight, converting all meat categories to retail weight ensured consistency across the data. This study followed the standardized conversion factors provided by the Agricultural Outlook 2023–2032 [15] (Table A3). It is important to understand that the values for converting a carcass into edible RWE can differ depending on the region, chosen methodology, processing techniques, and the desired end product [15]. 

Furthermore, adjustments were necessary to approximate values attributed to the Russian Federation before 1992, extrapolated from the available data on the USSR. Specifically, the process involved aggregating the populations of 15 post-Soviet states in 1992. Subsequently, the share of the Russian population within these 15 post-Soviet states in 1992 was used as a reference point. This share served as a basis for estimating the Russian food supply quantity during the pre-1992 period, facilitating the harmonization of data spanning the USSR era and the following period of the Russian Federation.

(1) $${Food\;Supply\;Quantity\;of\;Russia}_{pre-1992}=\frac{{Population\;of\;Russia}_{1992}}{{Total\;Population\;of\;15\;Post-USSR\;States}_{1992}}\cdot {Food\;Supply\;Quantity\;of\;USSR}_{pre-1992}$$

Lastly, data on Indian production quantity and the number of animals slaughtered for meat of cattle were missing from 1991 onwards. These data were necessary to calculate country-specific conversion factors for the conversion of meat weight to the number of animals affected. The average yearly growth in conversion factors up to 1990 was applied to the conversion factors from 1991 onwards to compensate for the missing data points. 

To forecast future trends for the year 2030, an alternate data source was utilized. The OECD database provides data on meat consumption predictions that were used to predict animal lives affected for the year 2030, albeit with slightly different elements and categories compared to FAOSTAT. In instances where specific data points were not available in the OECD database, namely for the mutton and goat category as well as for Germany, France, and Italy, the following assumptions were made. The mutton and goat assumption was informed by the growth percentage that could be calculated using the sheep category available in the OECD database, which was assumed to apply to the mutton and goat category available from FAOSTAT. Additionally, the growth percentage for the European Union (which was assumed to be the best representation of growth) that was calculated with data from the OECD database for 2020 and 2030 was applied to the data for Germany, Italy, and France from FAOSTAT for the year 2020.

### 2.2. Data Analysis

The food supply quantity serves as an indicator of meat consumption, which can be directly obtained from the FAO Food Balance Sheets (FBSs) and is presented in kilograms per capita per year [13]. The food supply quantity, in this context, refers to the amount of a particular food product available for domestic consumption in a given year [16]. It considers the domestic supply quantity (production + imports − exports) while adjusting for changes in stocks and excluding quantities used for purposes such as feed, seed, non-food applications, or those lost during processing. The commodities that remain after adjusting for these diversions are referred to as the ‘food supply’ [13].

Given that the FAO furnishes the food supply quantity per capita data, this study operationalized its analysis by multiplying the per-capita data with the respective populations of the G20 countries also obtained from FAOSTAT, thereby yielding the food supply estimates for each country and each year. This number represents the average amount of food available for consumption per country. For this study, per-country data were preferred over per-capita data because they shed light on the actual number of individual animal lives affected, not just whether more or fewer animals are slaughtered per person for consumption. It is important to consider that food supply data as a measure of meat consumption do not directly account for food consumption but only for food available for consumption. Consequently, this method may lead to an overestimation of consumption [16]. Still, food supply data were specifically relevant to this study, as animals are affected regardless of whether their meat is consumed or wasted.

Concerning the number of individual animal lives affected, intricate calculations were made. The data on meat traded are presented in the form of meat weight measured in thousand tonnes (1000 t) rather than in terms of the actual number of animals slaughtered. Several key data points can be employed from FAOSTAT to still derive the number of animals slaughtered for meat consumption (Table 1) [13].

**Figure 1:**
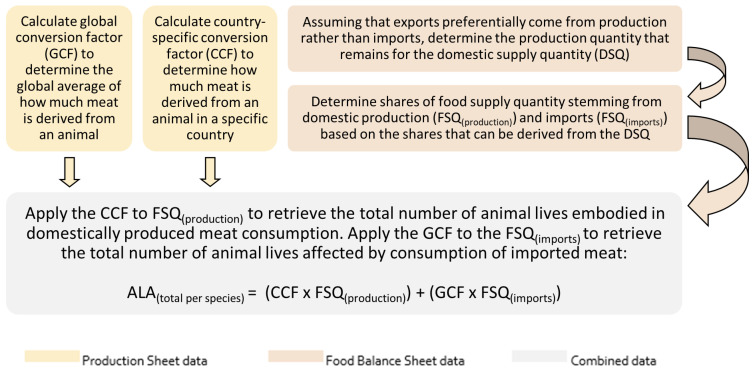
Multi-step process to estimate the number of animal lives affected.

Therefore, a multi-step process was followed (Figure 1), in which the streams of production (Production Sheet) and consumption (Food Balance Sheet) data were intertwined to retrieve the number of animal lives affected by meat consumption in each of the analyzed countries.

Thereafter, the framework created by Scherer et al. [17] was used based on the notion that the lives of species can be valued differently depending on their moral value (Table A4), which refers to their level of self-awareness and sense of time relative to that of a human. Commencing with a moral adjustment factor of 1 designated for humans, animals are assigned proportional moral adjustment factors—such as 0.027 for pigs—corresponding to their neuronal count or brain mass [17]. Following the method of Klaura et al. [18], which applies a simplified approach to the Scherer et al. [17] framework, the number of animal lives affected (ALA) were multiplied by the adjustment factors (MVF_j_) to account for the moral value of the animal lives in question:

Morally Adjusted ALA_(total per species)_ = ALA_(total per species)_ × MVF_j_. (2)

The outcome was a morally adjusted estimate of animal lives affected by meat consumption. Since such moral values are uncertain and applying them or not is a value-based choice, we presented unadjusted and adjusted estimates of the number of animal lives affected to provide more comprehensive insights.

To showcase the levels of growth in consumption, the number of animal lives affected, and the number of morally adjusted animal lives affected, growth factors were calculated by assessing the growth between the years 1961 and 2020, both the total values per country as well as the total values per animal category. A growth factor between 0 and 1 indicates a decrease in consumption, e.g., a growth factor of 0.9 means that the value in 2020 is 90% of the value in 1961. A growth factor of 1 means there is no change. Every growth factor greater than 1 indicates an increase in value. For example, a growth factor of 1.5 means that the value in 2020 is 150% of the value in 1961.

## 3. Results

### 3.1. Temporal Development in Meat Consumption

In examining the trends in meat consumption across the 19 countries constituting the G20, it is evident that there has been a substantial increase in meat consumed over the years. In 2020, approximately 194 billion kilograms of meat were collectively consumed within the 19 countries of the G20. Germany, the UK, and France are witnessing the least overall growth in consumption of the four meat categories combined, and Saudi Arabia, Korea, and China have the highest growth rate (Table A6).

**Figure 2:**
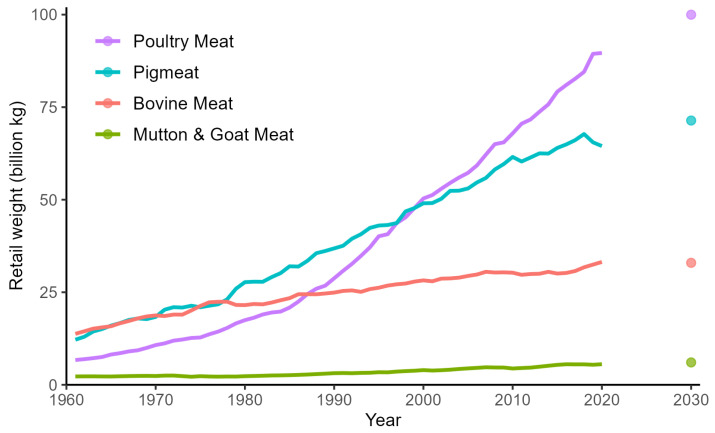
Trends in meat consumption per category from 1961–2020 and the predicted consumption in 2030.

**Figure A1:**
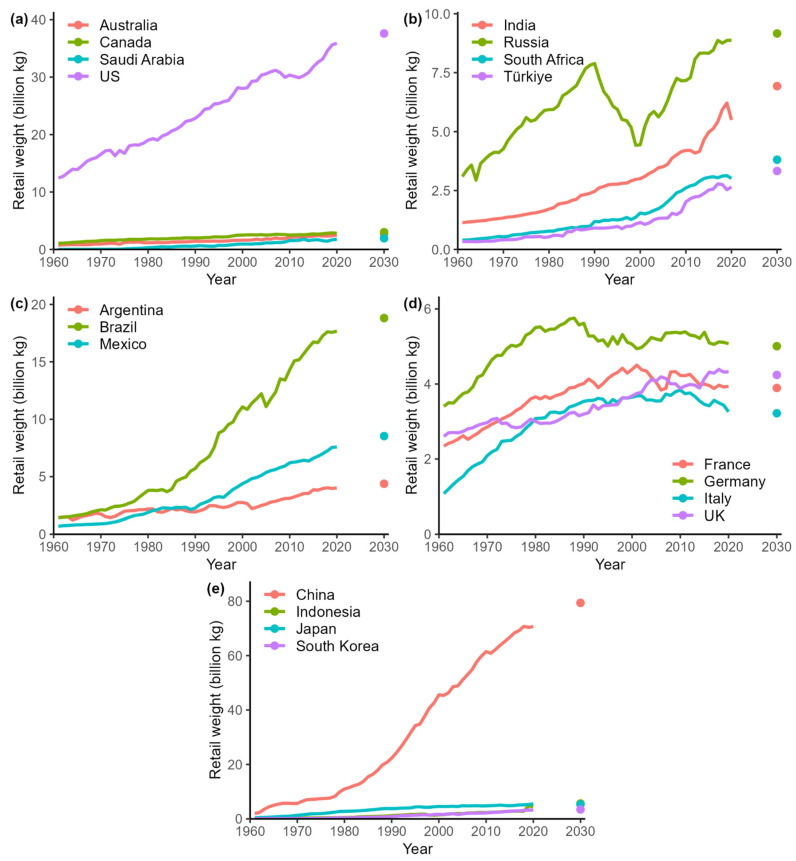
Total meat consumption per country. Panels (**a**–**e**) refer to different G20 country groups.

All categories showcase an increase in meat consumption between 1961 and 2020, albeit some categories substantially more than others (Figure 2). Poultry meat saw a staggering growth between 1961 and 2020, with a growth factor of 13, accounting for 46% of the total meat consumed in 2020. Particularly noteworthy is the exponential growth observed in countries such as Saudi Arabia, Brazil, and Korea. Pigmeat showed the second highest increase, except in Türkiye, where there was no consumption of pigmeat at all. Notably, Saudi Arabia emerged as the leader in pigmeat consumption, with an extraordinary growth factor of 34, indicating a surge in demand within this emerging economy. Bovine meat consumption also demonstrated a stable rise, albeit at a slower pace than pigmeat. Leading in bovine meat consumption was China, with a remarkable growth factor of 11. Interestingly, mainly Western countries, such as Germany and the UK, exhibited no increase in bovine meat consumption. Mutton and goat meat noticeably experienced the least growth, with a factor of 2.5 between 1961 and 2020 that accounted for just less than 3% of consumption in 2020. Several countries spread across different continents (e.g., Argentina, Japan, Australia, Russia, and the US) even witnessed a decline in consumption levels over the study period (Figure A1).

### 3.2. Temporal Development in Number of Animal Lives Affected

In assessing the animal lives affected between 1961 and 2020, the data reveal substantial changes in the number of animals affected over the six-decade period, whether looking at the categories or countries. 

**Figure 3:**
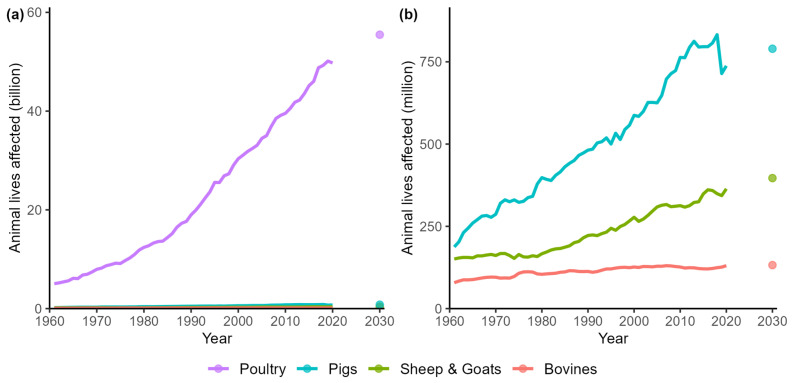
Trends between 1961 and 2020 in the total number of animal lives affected by meat consumption and the predicted numbers for 2030, (**a**) including all animal categories and (**b**) excluding poultry.

Poultry: The poultry industry had the most profound increase, with about 5 billion poultry animals affected in 1961 (Figure 3). By 2020, this number soared to about 50 billion, illustrating a remarkable surge in the number of poultry animals embodied in meat consumption. Saudi Arabia experienced the highest increase in the number of poultry animal lives affected, with an extreme growth factor almost reaching 300. This was followed by Indonesia and South Korea, with growth factors of about 70. The least growth in poultry animal lives affected was in France, Italy, and Germany.

Pigs: The number of pigs affected by meat production in 1961 was approximately 187 million, and by 2020, this number surged to about 738 million. China and South Korea had the highest increase in the number of pigs affected, with a growth factor exceeding 16. This was followed by Japan, with a growth factor of approximately 8, almost half the growth rate compared to the two leading countries. The UK, Canada, and the US witnessed the least growth in pig lives affected. Notably, there is a drop in the number of pigs affected from 2018 to 2019, which coincides with a drop in the number of animals affected in China.

Bovines: In 1961, approximately 79 million bovine animals were affected globally. By 2020, this figure rose to approximately 130 million, again indicating a considerable increase in the number of animals affected. Among the countries, China was again the leading country in terms of its growth rate in the number of animals affected, with a growth factor of over 60. This was (not so closely) followed by South Korea and Saudi Arabia, which witnessed growth factors of about 24 and 19.

Sheep and Goats: Despite a lower growth rate than most other categories, the total number of sheep and goats affected by meat consumption still increased from nearly 151 million in 1961 to approximately 364 million in 2020, reflecting a growth factor of approximately 2.4. China again experienced the highest growth (about 36), followed by South Korea and Saudi Arabia (growth factors of about 16 and 7). However, 10 out of 19 analyzed countries did not witness growth at all and even a decline in the number of sheep and goat lives affected. All growth factors per country and category can be found in Table A7.

**Figure 4:**
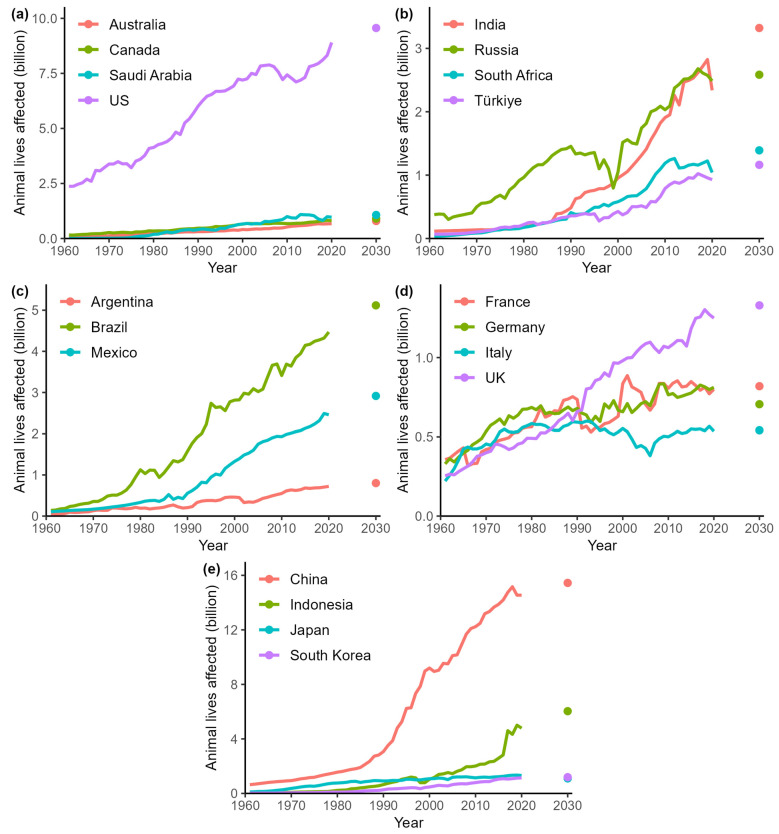
Animal lives affected by country. Panels (**a**–**e**) refer to different G20 country groups.

When comparing the growth factors of meat consumption with the growth factors of animal lives affected (Table 2), it can be observed that the latter lags behind the former. Despite substantial increases across all categories, the growth in the number of animal lives affected by meat production shows a slower pace of growth than that of meat consumption. This trend reverses when considering the overall growth factor of all animal categories together, where the number of animal lives affected grows faster than meat consumption. Importantly, these growth trends do not only apply at the level of the population but also when considering per-capita meat consumption (Table 2). The overall growth factor of the aggregated number of animal lives affected of all categories combined showed that France, Italy, and Germany experienced the least growth and Saudi Arabia, Indonesia, and South Korea the most (in that order; Figure 4).

### 3.3. Morally Adjusted Estimate of Number of Animal Lives Affected

**Figure 5:**
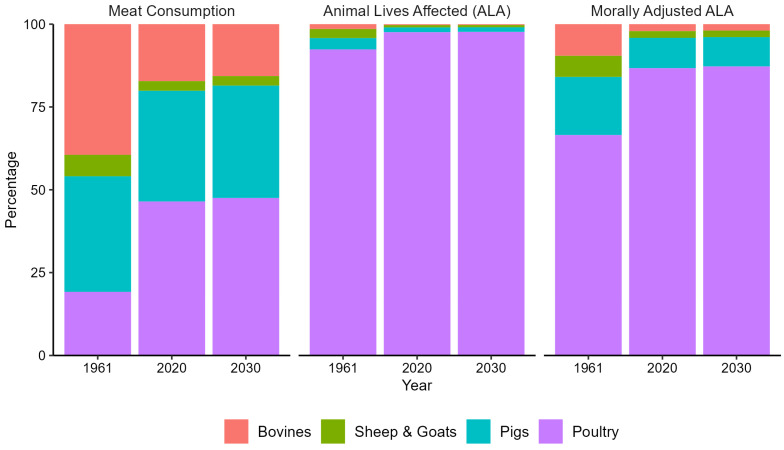
Percentage shares of animal categories in meat consumption, animal lives affected (ALA), and morally adjusted ALA.

The trends in the morally adjusted estimate of animal lives follow the same trajectory as animal lives affected and meat consumption: a steady increase for most animal categories and countries, with some exceptions for sheep and goats. However, when considering the moral value, the number of morally adjusted animal lives affected of poultry comes somewhat closer to that of the other categories. A notable finding emerges when examining the growth factors of the aggregated animal lives affected, either morally adjusted or not (Table 2). Although the growth factors remain consistent when considering each category separately, a noteworthy distinction arises regarding the overall growth when the moral value is applied. Since poultry animals are assigned a smaller moral value than the other animal categories, it takes up a smaller share of the total morally adjusted animal lives affected. The other categories show a lower growth rate on their own, but their shares of the total morally adjusted animal lives affected increases (especially for bovines). Consequently, the total growth factor of morally adjusted animal lives affected decreases as compared to the total animal lives affected. When comparing the overall development of all three items (meat consumption, animal lives affected, and morally adjusted animal lives affected) between 1961 and 2020, this change in the contributions of the different animal categories to the aggregated values is clearly visible (Figure 5).

### 3.4. Future Trends: 2030

Looking ahead to the year 2030, predictions for meat production and consumption reveal insights into the future landscape of the industry across different animal categories. When looking at the number of animals affected, poultry emerges as the category experiencing the biggest overall increase in the number of animals affected during this period (Figure 2). Bovines showcase the least overall increase in the number of animals affected. Expectations concerning consumption are largely similar except that bovine meat consumption slightly reduces between 2020 and 2030 (Figure 2). When comparing predictions for meat consumption and animals affected amongst the countries (Figure 4 and Figure A1), there is a lot of overlap. Altogether, China appears prominently in all categories, with substantial increases expected in both meat consumption and animals affected. Similarly, countries such as the United States and Brazil, which are forecasted to experience notable growth in meat consumption, also demonstrate considerable impacts on the number of animals affected. The only countries that exhibit a decrease in both meat consumption and animal lives affected between 2020 and 2030 are Japan and Germany. Although the UK, Italy, and France also reduce their meat consumption, the number of animals affected continues to increase until 2030. The morally adjusted animal lives affected follows the trend of the animals affected; the number of morally adjusted poultry lives affected continues to grow at a faster rate than the other categories. However, while the share of poultry within the total number continues to increase, the overall growth in the number of morally adjusted animal lives affected increases more slowly.

## 4. Discussion

### 4.1. Trends Explained

The countries within the G20 are increasingly consuming more meat, and the number of animals affected is increasing, whether per category or the total aggregated values. The growth factors for per-capita consumption illustrate that while population growth contributes to overall consumption trends, it does not fully account for all observed increases in consumption (Table 2 and Table A6).

Disparities in growth rates among different animal categories, with poultry experiencing the highest increase and bovines the least, can be influenced by a combination of factors. Valceschini [19] highlights the aspects that explain the increasing interest in poultry over other meat types: poultry has a highly competitive price compared to other meats, faces the least cultural and religious obstacles to its consumption, and provides dietary and nutritional benefits (high protein, low fat) over other meats. Out of all red meats, pigmeat is seeing the highest growth factor in consumption. One explanation for this, as Fowler [20] concisely summarized, is that “pigs have the misfortune to be physiologically amenable to a high degree of intensification”. They have high productivity (25–30 offspring/sow per year) and a potential growth rate of nearly one kilogram per day from birth to slaughter [20]. Another explanation is that bovine and mutton and goat meat are lagging due to the insufficient use of production development opportunities [21]. Despite an increase in the production of beef, mutton, and goat meat, their share of the total consumption of meat is decreasing as a consequence of the high growth rates of poultry and pigmeat [21].

There are also several regional disparities in the consumption trends. Overall, we see exponential growth in Saudi Arabia (Table A6 and Table A7). Saudi Arabia is a country that has seen considerable economic and population growth over the past few decades [15]. Additionally, the Saudi Arabian government has taken various initiatives to boost domestic meat production instead of relying on its current import-dominant meat supply [22]. While the steep growth in meat consumption in emerging countries is worrisome by itself, it can also create lock-in effects that make it difficult to reverse such trends [23].

The countries that see the least growth in both meat consumption and animals affected (e.g., Germany, the UK, France) have also experienced population growth, albeit more slowly and stably, but various socio-economic factors make that growth in meat consumption and animals affected stagnate in these Western countries: a cultural and dietary shift towards more vegetarian and vegan options, increasing awareness of health and environmental concerns related to the meat industry, more governmental policies and incentives to shape consumer behavior, technological advancements leading to improved plant-based meat alternatives, and increased production efficiency [24].

An unusual interruption of the general trend can be seen in the case of Russia. Consumption declined when the USSR was dissolved and the Russian Federation was founded in 1991 (Figure A1b). Thereafter, Russia experienced a dip in meat imports due to the Russian economic crisis, which hit its peak in late 1998, making imports more expensive for Russian consumers. This led to a proportionate dip in the total number of animals affected for that period, as the number of slaughtered animals embodied in imported meat plummeted (Figure 4). Meat imports recovered from 2000 onwards to almost pre-crisis levels in 2010 as the ruble began to gain value again compared to other currencies [25]. Similarly, a decline in pigmeat consumption can be noted in 2018–2019. This dip can primarily be attributed to the outbreak of the African Swine Fever (ASF) [26]. ASF is a highly contagious viral disease, which led to the widespread culling of pigs to control its spread [27]. This particularly took place in major pork-producing countries, such as China and Southeast Asian countries [28]. The massive reduction in pig populations resulted in a decrease in pork supply, causing a drop in consumption. Additionally, disruptions in pork production and the increased pork prices due to ASF further discouraged consumption, prompting consumers to shift to alternative meats or reduce their overall meat intake [28]. This health crisis, similar to COVID-19, underscored the vulnerability of meat supply chains to disease outbreaks, influencing global consumption patterns. 

### 4.2. Proportionate Growth

As the consumption of meat increases, there exists an expected correlation wherein the demand for meat necessitates a proportional increase in the number of animals slaughtered. This ‘logical’ correlation is exactly what this study aimed to interrogate and challenge. The results show that a difference exists between considering the proportionate growth per animal category between 1961 and 2020 and the proportionate growth of all animal categories combined during that same time. Other factors beyond consumption levels thus play a role in explaining this nuanced relationship between meat consumption and its impact on animals. 

When examining each meat category separately, the higher growth in meat consumption as compared to animals affected (Table 2) indicates an increased production efficiency; fewer animals need to be slaughtered to meet the same demand. Examples of increased efficiency include letting the animals grow fatter so that more meat can be derived from one animal [29] or feeding antibiotics to the animal to reduce losses [30]. It can be questioned whether such methods to increase production efficiency are desirable, as they raise a lot of additional animal welfare issues (e.g., heart diseases and infertility amongst livestock animals) and human health concerns (e.g., antimicrobial resistance as a consequence of antibiotic-resistant bacteria being transmitted to consumers through meat) [29,30]. Sheep and goats see a much smaller difference in the growth rate for animals affected and meat consumption. A possible explanation is that mutton and goat meat production occurs at a higher share in developing countries than the other meat categories [13], and they face high animal losses due to diseases that cause abortion and reduced fertility [31].

When we consider the combined animal categories, more animals are getting affected in total over time in proportion to how much more meat we consume. When aggregating the values of all categories, the combined total (and thus the net effect of all categories) reflects the cumulative impact of all categories on animals affected and meat consumption. Such an aggregation reveals broader trends that are not directly apparent in the analysis of each category individually but are caused by a ‘consumption shift’ from one category to the other. For example, if there is a shift towards more poultry meat consumption and less consumption of bovine meat, it impacts the overall balance between meat consumption and the animals affected. Despite being one of the most environmentally efficient and least resource-intensive (due to a high feed-to-protein meat conversion) meat, poultry meat is one of the least efficient in the use of animals per amount of produced meat compared to other meat types [15]. Thus, shifting consumption to a category such as poultry, which requires a lot of slaughter for the same output of meat, results in an overall less efficient meat production and, thus, more animals in total being affected. The production efficiency increases within the individual categories are small compared to the differences between the categories (Table 2). 

The morally adjusted estimate of animal lives embodied in meat consumption shows the same growth trend per category as animals affected, but the aggregated growth factor is lower than that of animals affected. This can be explained by the share of the poultry category in the total morally adjusted animal lives affected, as it has the lowest moral value out of the four categories (Table A4). This means that while the share of poultry in the total number of animals affected is increasing, their sentience compared to that of pigs, bovines, or sheep and goats results in a slower growth of the total number of morally adjusted animal lives embodied in our consumption. The animals’ sentience reflected in the moral value is linked to their capacity to suffer, and a higher moral value is, therefore, assumed to entail higher risks for animal welfare [32]. The finding underscores the importance of ethical considerations in assessing the actual impact on animal welfare, revealing a disparity between the raw numerical growth and the more nuanced understanding afforded by moral frameworks.

### 4.3. Future Predictions: Toward 2030

The same factors that have been stimulating increased meat consumption and animals affected up to 2020 will continue to do so in the future: growing populations, rapid urbanization, and rising incomes are driving increased demand, and production efficiency and technological modernization of the livestock industry are expected to increase availability for the consumer [15]. However, in developed countries such as Germany, France, and the UK, ethical concerns and political interventions may stagnate consumption patterns and the number of animals affected [15]. Such countries could play a role in setting an example for the future trajectory of the global meat industry and its associated effects on animal welfare. However, a proper example would require these countries to reverse trends and noticeably decrease the number of animals affected, whereas they are currently, at best, showcasing a stagnation of upward trends.

We should also be wary of such countries ‘exporting’ the problems associated with high-intensity livestock farming to the Global South. For example, the high amounts of manure and wastewater generated and often poorly managed are a concern for the environment and public health [33]. Increased livestock densities and antibiotic use increase the risk of infectious disease emergence, while access to health care is limited in developing countries [34]. Moreover, if animal feed competes with human food for land resources, it contributes to food insecurity [35]. According to Westhoek et al. [36], the European Union already imports four-fifths of the protein-rich feed required for livestock production. 

Emerging economies present diverse scenarios for future meat consumption and its impact on animals due to more dynamic import/export structures and oftentimes less stable political systems. For instance, India relies heavily on domestic production and may face stable growth, whereas countries such as Saudi Arabia rely more on imports. This can lead to them facing challenges in achieving self-sufficiency and ensuring food security amidst fluctuating import policies and shifting consumer preferences. Despite overall consistent trends, challenges related to disease outbreaks (e.g., COVID-19), economic crises, and environmental and resource constraints may impede growth [15].

### 4.4. Barriers Affecting Animal Lives

Given the central role of consumers within this study and how they are driving demand for meat and thereby affecting animal lives, it is crucial to clarify the barriers hindering efforts to reduce meat consumption and the number of animal lives affected. 

One major factor is the physical and emotional disconnect between consumers and farmed animals, which contributes to low consumer awareness regarding the suffering experienced by these animals [8]. This disconnect makes it easier for consumers to overlook the harsh realities of meat production. In addition to this, consumers also dissociate meat from its animal origins, which limits their moral concern for farmed animals and reduces their willingness to make dietary changes. The psychological distance diminishes empathy and moral consideration [2,37]. Some consumers deny that animals possess sentience, further diminishing moral concern for their welfare and allowing them to rationalize meat consumption without confronting ethical implications [38,39].

Various justifications are employed to rationalize meat consumption, including beliefs about the necessity of meat for nutrition, tradition, and taste preferences. These justifications help alleviate cognitive dissonance associated with the ethical concerns for eating animals [40,41]. The pleasure derived from eating meat is another significant barrier to reducing meat consumption, as the sensory enjoyment of meat can overshadow ethical considerations [42]. 

Moreover, demographic factors such as gender, race, ethnicity, and education level influence consumption patterns. For instance, men tend to consume more meat than women. Cultural associations between meat consumption and masculinity play a role in sustaining high levels of consumption. Meat is often perceived as a symbol of strength and masculinity, discouraging men from adopting plant-based diets [43]. Furthermore, younger individuals are more likely to adopt plant-based diets than older generations, and higher levels of education are often associated with lower meat consumption [44,45]. Lastly, as argued before, ethnic and cultural backgrounds can shape dietary habits, with some cultures having a stronger preference for meat consumption [46]. Understanding these demographic influences is crucial for developing targeted governmental interventions to reduce meat consumption. 

### 4.5. Governance Implications

In examining the complex interplay between meat consumption trends and the animals affected, it becomes evident that legislative interventions play a pivotal role in shaping the future trajectory of the livestock industry. This section will follow the hierarchical “Three R’s” principles proposed by Scherer et al. [17] to explore opportunities to reverse trends in the number of animals affected by meat consumption or mitigate the effects themselves: replace, reduce, and refine.

#### 4.5.1. Replace and Reduce: Stimulating Shifts toward More Plant-Based Diets

The “replace” approach implies a full replacement of animal-based products with plant-based alternatives, whereas the “reduce” approach involves reducing the consumption of animal-based products but not necessarily eliminating them entirely [17]. A practical example of replacement was showcased at the engineering faculty of the Technical University Delft, which fully eliminated meat and fish from its cafeteria [47]. A similar example was the citizens’ initiative introduced by Sentience Politics (a Swiss political organization) in Berlin that would require all schools and the town hall to offer a vegan option every day [48]. These case examples are all bottom-up initiatives. Top-down strategies could support such bottom-up initiatives to stagnate the number of animals affected by consumption. Examples that Wellesley et al. [49] provide of such top-down legislative measures in support of plant-based diets include implementing taxes or subsidies, setting regulations on food labeling, restricting the sale of meat and other animal-based products, and establishing public procurement guidelines. Bonnet et al. [6] add that legislative intervention should involve promoting alternatives to traditional meat production, such as plant-based and lab-grown alternatives. This way, the bottom-up approaches are more easily realized due to top-down support.

#### 4.5.2. Reduce: Stimulating Shifts toward Different Meat Categories

Another strategy within the “reduce” approach is to shift from meat categories with higher animal slaughter ratios, such as poultry, to those with lower ratios, such as bovine meat or pork. The animal slaughter ratio refers to how many individual animal lives are required to produce a given amount of meat for consumption. Promoting a dietary shift toward meat with a lower animal slaughter ratio could potentially lower the total number of animals slaughtered for a given level of meat consumption, but it is not a very popular legislative strategy. Deciding on legislation in support of such a transition comes with considerable trade-offs. While shifting from poultry to bovine meat or pigmeat reduces the number of individual animals affected, it raises additional environmental concerns. Beef and pork production generally entail higher greenhouse gas emissions than poultry [50]. Additionally, these meat categories’ production requires more feed inputs per unit of meat output, placing greater strain on land and resource utilization [35]. Furthermore, there are ethical considerations to address. While poultry production involves higher numbers of individual animals, cattle and pigs are often perceived as having more complex cognitive and emotional capacities, raising ethical concerns about promoting red meat diets [51]. However, the moral value attempts to account for such different capacities, and it seems insufficient to compensate for the lower yield [17].

#### 4.5.3. Refine: Improving Animal Welfare Standards

Recognizing that meat consumption is unlikely to reach zero in the foreseeable future, it becomes imperative to additionally prioritize the welfare of animals. The “refine” approach focuses on improving the conditions and welfare of those animals that remain in livestock farming systems. International organizations, such as the World Organisation for Animal Health (OIE), play a crucial role in refining animal welfare. The OIE’s Terrestrial Animal Health Code provides guidelines for various aspects of animal welfare, including intensive farming practices [52]. Moreover, collaborative initiatives, such as the Welfare Quality Project, offer standardized frameworks for assessing animal welfare, facilitating the development of legislation aligned with international principles [53]. However, these again are examples of bottom-up initiatives; governments must incorporate these standards in their institutional framework for animal welfare legislation to achieve successful top-down implementation. Improved legislation on animal welfare not only fosters ethical treatment but can also yield economic advantages. By enhancing basic health and biological functioning, such as minimizing disease, injury, malnutrition, and mortality rates, production efficiency is improved [53]. After the initial investments to improve animal welfare, the increased production efficiency reduces production costs.

#### 4.5.4. Challenges and Opportunities

A big challenge in legislation lies in policy discussions surrounding meat consumption, which often center on developed countries, while implications extend globally. Developing international norms and standards is necessary to help promote reduced meat consumption in both developed as well as developing countries, in some of which consumption levels are also already high [49], whereas they are currently lagging in terms of animal-friendly policies. There is also the possibility that developing countries learn from developed countries and perhaps hop on the plant-based trends sooner than the developed countries [6].

Despite attempts at improving animal welfare legislation by mainly implementing legislation on transport and slaughter methods, legislation has a long way to go when it comes to reducing the number of animals affected. Governments often hesitate to intervene with more drastic measures due to fears of economic repercussions and public resistance, which is why current legislation in most countries is not achieving the necessary results. Also, governments have to deal with complex trade-offs and face the challenge of designing policies that balance competing interests of environmental, health, and ethical implications while effectively reversing meat consumption trends. Governments seem trapped in a cycle of inertia; they worry about negative consequences of intervention, whereas the lack of intervention may hide the problem from the public, whose lack of awareness, in turn, avoids putting pressure to intervene [49]. Altogether, while promoting plant-based diets and better animal welfare legislation are commendable goals, drastically progressive legislation is necessary to reverse the increasing trends in the number of animals affected. For now, most people want to eat cheap meat without a guilty conscience about the environment or the animals. However, social norms are changing progressively, and regulating meat may be increasingly accepted by society, as well as dietary changes and associated policies [6].

### 4.6. Research Outlook

Understanding the interplay of social, economic, political, and demographic factors can provide valuable insights into the disparities observed in growth rates among different animal categories. 

The seemingly large numbers of animal lives affected that this study presents are still an underestimation. As Klaura et al. [18] show, there are losses of animal lives before slaughter, which are missing in the estimations of this study. The study is also limited to the four broad meat categories. Future studies should strive to incorporate the analysis of animals embodied in the consumption of fish, eggs, and dairy products, as these sectors are too often neglected in research despite the huge global demand for them. For instance, evidence shows that newborn calves in dairy industries are often killed at birth or when very young [6]. 

Also, the analysis in this study is limited to quantifying the number of animals affected by meat consumption. This number has been found to be a decisive factor for the overall animal welfare loss [17], but it lacks insights into the quality of the animals’ lives. The conditions under which the animals are affected can vary considerably due to differences in legislation, farming practices, transportation methods, and slaughter techniques across various regions and animal species. For future research, it would be valuable to couple these results on how many animals are affected with studies on how animals are affected to provide a more comprehensive understanding of animal lives affected.

Additionally, future research endeavors should identify and aim to overcome the existing limitations in terms of measuring animal welfare and successful policy measures. The fundamental question of comparing animal welfare, both within and across species, may become a focal point of future academic and policy discourse. Efforts to mitigate cultural and geographical biases in meat consumption, animal welfare, and policy analyses are paramount, necessitating the integration of diverse perspectives and locally contextualized data. The countries chosen for this analysis represent both developed and emerging economies and at least one country from each continent. This ensures a wide representation of countries differing in economic, cultural, and governmental characteristics. However, scholars and governmental actors should strive to improve data quality and availability from different countries, especially in regions where reliable information is lacking, to ensure more accurate and representative estimations. Additionally, opportunities and barriers for legislative changes across the full policy cycle deserve further exploration. Strategies to improve animal welfare require interdisciplinary collaboration, for example, among animal welfare scientists, economists, and social scientists, to ensure their effectiveness [54].

Future predictions are inherently uncertain. As Bamberger et al. [55] also state, projections depend on many assumptions about how social, economic, and environmental systems may change. This study uses the OECD/FAO Agricultural Outlook 2023–2032, but predictions for crucial milestone years, such as 2030, become more accurate the closer we get to them [55]. Therefore, a reiterative research approach must be taken to predict future trends, and even then, dynamic factors, such as unforeseen events and crises, such as COVID-19, demand adaptation and flexibility in terms of research methods and policy frameworks. 

The approach to this study is partly generalizable in the sense that it might also be useful for future research on animals embodied in production and consumption and especially in extending it to other countries or other animal products.

## 5. Conclusions

In the realm of global food systems, meat consumption stands as a pivotal issue with far-reaching implications for animals. This study has delved into the intricate web of factors that influence trends in the number of animals affected within the G20 countries, with meat consumption as the main driver. The analysis has revealed key insights that demand attention as well as action. 

The first of such key insights pertains to the temporal development of global meat consumption. By tracing historical trends and projecting future trajectories, it becomes evident that the demand for meat is on a steady rise, fueled by factors such as population growth, rising affluence, and shifting dietary preferences. This upward trajectory poses significant challenges in terms of animal welfare, necessitating a reevaluation of current consumption patterns and a reorientation towards more sustainable food choices.

The primary objective of this research study is to quantify the number of animals affected by meat consumption trends within the G20 countries. This quantification has provided a stark reminder of the individual lives at stake in meat consumption, which is, in total, even growing at a faster rate than meat consumption due to shifts in consumption from animals that have a high conversion rate from animal to meat to animals that have a lower conversion rate. As highlighted by the results, the sheer scale of animal slaughter for human consumption is staggering and underpins the ethical considerations of our diets. 

Legislation and policy measures play a crucial role in shaping the landscape of meat consumption and its impact on animals. The existing literature on what makes for successful legislative frameworks has revealed a necessity for governmental intervention and support for bottom-up initiatives. By advocating for strong regulations, promoting plant-based diets, and fostering a culture of ethical consumption, policymakers can steer food systems toward a more sustainable and animal-friendly future.

The implications of this study extend beyond academic discourse to practical applications and real-world implications. By quantifying the individual animal lives affected by meat consumption trends, this study aims to provoke reflection, inspire action, and pave the way for a future where food choices are guided by principles of empathy, sustainability, and respect for all living beings. Thus, instead of centering our choices around humanity and viewing things solely from a human perspective, we can urge ourselves to consider the animal perspective within the meat industry, thereby broadening our understanding and fostering greater empathy toward the lives affected by our dietary choices.

## Figures and Tables

**Table 1:** Items used for calculating conversion factors. Source: [13].

Item	Definition	Data Type
Production Quantity	Offers insights into the total meat production within a specific country in CWE.	Production-Based Data
Producing Animals/Slaughtered	Information on the number of animals raised or slaughtered for meat production within a given region.	Production-Based Data
Export and Import Quantity	Quantities of meat exported from and imported into a country, reflecting the consumption dynamics.	Supply Data

**Table 2:** Growth factors 1961–2020 of the G20 countries per animal category.

Category	Meat Consumption (G20 Countries)	Meat Consumption (per Capita)	Animal Lives Affected (ALA)	Morally Adjusted ALA
Bovines	2.4	0.98	1.7	1.7
Pigs	5.3	1.79	3.9	3.9
Poultry	13.4	6.99	9.8	9.8
Sheep and Goats	2.5	0.38	2.4	2.4
Total	5.5	2.04	9.3	7.6

**Figure A2:**
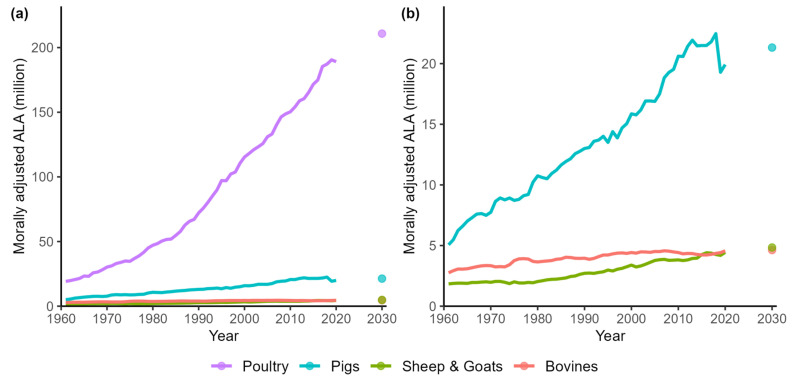
Trends between 1961 and 2020 in the total number of morally adjusted animal lives affected (ALA) by meat consumption, (**a**) including all categories and (**b**) excluding poultry, and the predicted number of morally adjusted ALA by 2030.

**Figure A3:**
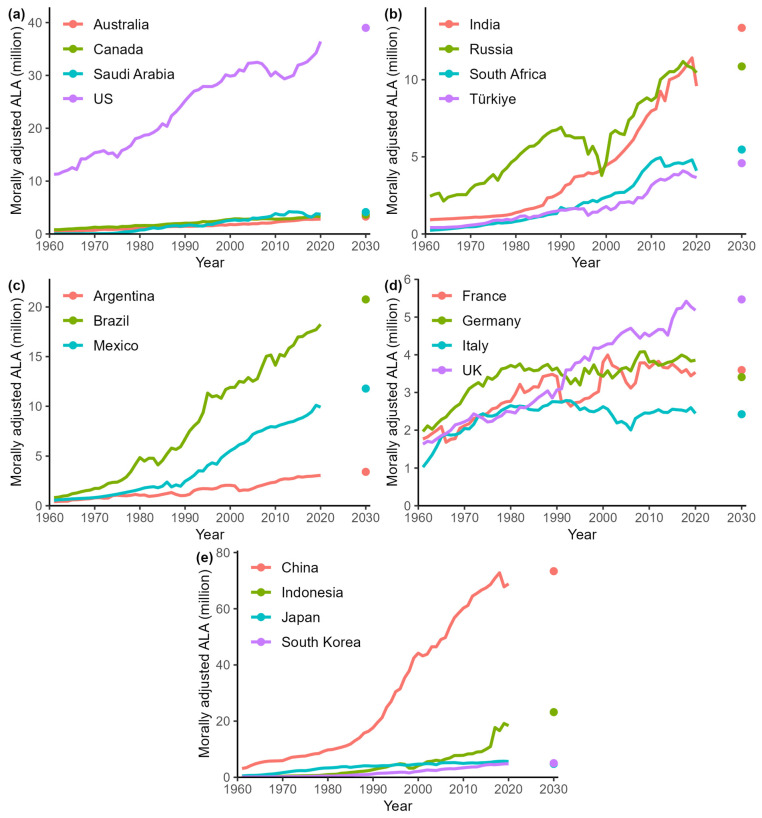
Total morally adjusted animal lives affected (ALA) per country. Panels (**a**–**e**) refer to different G20 country groups.

## Data Availability

The original contributions presented in the study are included in the Supplementary Material.

## Supplementary Materials

The following supporting information can be downloaded at: https://www.mdpi.com/article/10.3390/ani14111662/s1, an Excel file with the data behind all figures.

## Appendix A

**Table A1:** Countries featured in the analysis. Sources: FAO country codes and country lists from FAOSTAT [13]; region assignment from Klaura et al. [18].

FAO Country Code	Country	Region
32	Argentina	Latin America
36	Australia	North America and Oceania
76	Brazil	Latin America
124	Canada	North America and Oceania
159	China	Industrialized Asia
250	France	Europe
276	Germany	Europe
360	Indonesia	South and Southeast Asia
365	India	South and Southeast Asia
380	Italy	Europe
392	Japan	Industrialized Asia
484	Mexico	Latin America
410	Republic of Korea	Industrialized Asia
643	Russian Federation	Europe
682	Saudi Arabia	North Africa, Western and Central Asia
710	South Africa	Sub-Saharan Africa
792	Türkiye	North Africa, Western and Central Asia
826	United Kingdom	Europe
840	United States of America	North America and Oceania
810	USSR ^1^	Europe and Northern and Central Asia

^1^ USSR data was used for estimating the share of Russian production and import and export quantity between 1961–1991.

**Table A2:** Meat categories and their descriptions. Source: [13].

Category	Default Composition FAOSTAT (Including FAOSTAT Code)
Bovine meat	867 Meat, cattle, 870 Meat, cattle, boneless (beef and veal), 872 Meat, beef, dried, salted, smoked, 873 Meat, extracts, 874 Meat, beef and veal sausages, 875 Meat, beef, preparations, 876 Meat, beef, canned, 877 Meat, homogenized preparations, 947 Meat, buffalo
Pigmeat	1035 Meat, pig, 1038 Meat, pork, 1039 Bacon and ham, 1041 Meat, pig sausages, 1042 Meat, pig, preparations
Poultry meat	1058 Meat, chicken, 1060 Fat, liver prepared (foie gras), 1061 Meat, chicken, canned, 1069 Meat, duck, 1073 Meat, goose and guinea fowl, 1080 Meat, turkey
Mutton & Goat meat	977 Meat, sheep, 1017 Meat, goat

**Table A3:** Conversion factors from meat expressed in carcass weight equivalent to boneless retail weight equivalent. Source: [15].

Species	Conversion Factor
Bovine Meat	0.67
Pigmeat	0.73
Mutton and Goat Meat	0.66 ^1^

^1^ The Agricultural Outlook 2023–2032 [15] only provides the conversion factor for sheep, and the assumption was made that the factor remains the same for goat meat.

**Table A4:** Moral adjustment factors to calculate morally adjusted estimates of animal lives affected.

Species	Moral Adjustment Factor	Source
Bovines	0.035	[17]
Pigs	0.027	[17]
Poultry	0.0038 ^1^	[17]
Sheep and Goats	0.0122 ^2^	[56]

^1^ Scherer et al. [17] provide the moral adjustment factor for chicken, and the assumption is made that the factor remains the same for other animals in the poultry category. ^2^ Scherer et al. [56] provide the moral adjustment factor for sheep, and the assumption is made that the factor remains the same for goats.

**Table A5:** Elements used to make calculations. Sources: [13,15].

Data Source	Code	Element	Unit	Description
Food Balance Sheet	511	Total Population—Both sexes	1000 No	Total population—both sexes
	5511	Production	t	Total meat production (from both commercial and farm slaughter. Production of beef and buffalo meat includes veal; mutton and goat meat includes meat from lambs and kids; pigmeat includes bacon and ham in fresh equivalent. Poultry meat includes meat from all domestic birds and refers, wherever possible, to ready-to-cook weight.
	5510	Production	1000 t	Total meat production (from both commercial and farm slaughter. Poultry meat includes meat from all domestic birds and refers, wherever possible, to ready-to-cook weight.
	5611	Import Quantity	1000 t	Total meat imported
	5911	Export Quantity	1000 t	Total meat exported
	5301	Domestic Supply Quantity	1000 t	Production + imports − exports + changes in stocks (decrease or increase). It refers to the supply for domestic utilization.
	645	Food Supply Quantity (kg/capita/yr)	Kg	Production + imports – exports, but adjusted for changes in stocks and excluding quantities used for purposes like feed, seed, non-food applications, or those lost during processing. The total food available for consumption.
Production Sheet	5320	Producing Animals/Slaughtered	An	The total number of animals slaughtered for bovines, pigs, mutton and goats.
	5321	Producing Animals/Slaughtered	1000 An	Total number of animals slaughtered for poultry
OECD/Agricultural Outlook 2023–2032	--	CWE to RWE conversion factor	1000 t to kg	Carcass weight equivalent to boneless retail weight equivalent

**Table A6:** Growth factors per country—meat consumption between 1961 and 2020. Sorted from highest to lowest in column “Total”. The colors classify the growth factors by magnitude. Green: <1, yellow <2, orange <5, light red <10, dark red >=10.

Country	Bovine Meat	Mutton & Goat Meat	Pigmeat	Poultry Meat	Total	Total (per Capita)
Saudi Arabia	35.30	8.17	0.00	465.74	87.42	10.21
Republic of Korea	41.91	24.77	31.61	71.44	41.98	20.90
China	112.04	53.23	32.46	32.74	35.62	16.51
Indonesia	4.93	3.84	2.59	68.27	18.01	6.02
Japan	8.17	0.72	13.19	21.50	14.13	10.53
Brazil	5.72	3.24	5.76	79.52	12.40	4.36
Mexico	5.39	3.45	5.69	33.44	10.96	3.47
Türkiye	12.32	0.37	0.00	25.79	7.95	2.67
South Africa	2.79	1.34	4.45	59.50	7.56	2.30
India	1.51	2.34	2.15	46.57	4.84	1.59
Australia	2.20	0.56	5.73	24.83	3.41	1.39
Italy	1.27	1.11	4.98	4.36	3.02	2.53
United States of America	1.63	0.51	1.96	6.34	2.89	1.63
Russian Federation	1.30	0.42	2.19	10.72	2.87	2.20
Argentina	1.21	0.44	3.57	48.46	2.78	1.29
Canada	1.55	1.36	1.69	6.00	2.64	1.28
France	1.01	1.48	1.66	2.90	1.68	1.21
United Kingdom	0.88	0.43	1.20	6.58	1.66	1.31
Germany	0.83	1.46	1.33	4.15	1.49	1.32

**Table A7:** Growth factors per country—animal lives affected (per category and total) and the morally adjusted ALA (total) between 1961 and 2020. Sorted from highest to lowest in column “Total”. The colors classify the growth factors by magnitude. Green: <1, yellow <2, orange <5, light red <10, dark red >=10.

Country	Bovines	Pigs	Poultry	Sheep & Goats	Total	Total Morally Adjusted ALA
Saudi Arabia	19.35	0.00	289.77	6.82	230.44	156.65
Indonesia	2.18	2.50	70.37	4.18	66.44	53.66
Republic of Korea	23.52	16.39	68.68	15.59	65.08	50.70
Brazil	4.49	4.23	35.01	3.00	32.23	22.20
South Africa	1.54	2.92	43.25	0.53	32.10	17.32
Mexico	3.60	4.13	25.11	0.02	23.60	17.97
China	65.17	16.90	22.67	35.69	22.61	21.97
India	0.47	2.15	27.22	2.34	20.15	10.44
Argentina	1.04	3.00	27.76	0.44	18.60	7.45
Türkiye	3.24	0.00	17.52	0.22	13.50	8.72
Japan	4.55	8.03	12.76	0.66	12.47	11.38
Australia	1.17	3.36	17.25	0.41	11.29	5.93
Russian Federation	0.66	1.39	7.59	0.35	6.59	4.28
Canada	0.87	1.19	5.26	1.13	5.04	4.05
United Kingdom	0.50	0.85	5.77	0.39	4.92	3.17
United States of America	0.98	1.26	3.87	0.50	3.76	3.23
Germany	0.49	1.27	2.60	1.32	2.46	1.96
Italy	0.84	4.71	2.44	0.69	2.42	2.39
France	0.59	1.61	2.30	1.29	2.24	2.00
